# CTRP3 Stimulates Proliferation and Anti-Apoptosis of Prostate Cells through PKC Signaling Pathways

**DOI:** 10.1371/journal.pone.0134006

**Published:** 2015-07-28

**Authors:** Qi Hou, Jinyan Lin, Wentao Huang, Maoyin Li, Jianhua Feng, Xiangming Mao

**Affiliations:** 1 Department of Urology, Longgang District Central Hospital, Shenzhen, China; 2 Health management center, Nanfang Hospital, Southern Medical University, Guangzhou, China; 3 Department of Urology, Third Affiliated Hospital, Sun Yat-sen University, Guangzhou, China; 4 Department of Urology, Peking University Shenzhen Hospital, Shenzhen, China; Thomas Jefferson University, UNITED STATES

## Abstract

C1q/TNF-related protein-3 (CTRP3) is a novel adipokine with roles in multiple cellular processes. However, little is known about its function in prostate cells. This study investigated the effects and mechanisms of CTRP3 in prostate cells. We first generated and purified CTRP3 protein in HEK 293T cells. Proliferation of RWPE-1 prostate cells was evaluated by MTT analyses under treatment with different concentrations of CTRP3 for various exposure times. The results revealed maximum enhancement of proliferation with 10 μg/mL CTRP3 for 72 h. Cell apoptosis and cell cycle were determined by TUNEL staining and flow cytometry analysis. TUNEL assay showed decreased TUNEL-positive cells in RWPE-1 prostate cells treated with CTRP3, and flow cytometry showed significantly decreased apoptotic cells upon CTRP3 treatment (treated cells, 8.34±1.175 vs. controls, 20.163±0.35) (*P* < 0.01). Moreover, flow cytometry analysis also showed a significant decrease of cells in the G1 phase and an increase of cells in the S and G2 phase upon CTRP3 treatment (treated cells, 42.85±1.40 vs. control, 52.77±0.90; 28.41±0.57 vs. 23.49±1.13; 27.08±1.97 vs. 22.20±1.32, respectively) (all P < 0.05). Two-dimensional gel electrophoresis and mass spectrometry identified differentially expressed proteins, including cytokeratin-19, GLRX3 and DDAH1, which were upregulated in CTRP3 treated cells, and cytokeratin-17 and 14-3-3 sigma, which were downregulated. GLRX3, DDAH1 and 14-3-3 sigma were confirmed using western blot analysis. A PKC inhibitor, staurosporine, was used to inhibit PKC activity in CTRP3 treated RWPE-1 cells. Staurosporine completely abolished the CTRP3-induced increased phosphorylation of intracellular PKC substrates and CTRP3-stimulated effect by RWPE-1 cells. Our results provide the first evidence for a physiological role of the novel adipokine, CTRP3, in prostate cells. Our findings suggest that CTRP3 could improve proliferation and anti-apoptosis of prostate cells through protein kinase C signaling pathways.

## Introduction

C1q tumor necrosis factor-related proteins (CTRPs) are members of the highly conserved family of adiponectins. Each of the 15 known members (CTRP1–CTRP15) exhibits homologous structures composed of four distinct domains: a signal peptide at the N terminus, a short variable region, a collagenous domain and a C-terminal globular domain [1]. Members of the CTRP family take a part in diverse systems, such as the immune, endocrine, skeletal, and vascular system.

CTRP3 (also known as cartducin, cartonectin) was first reported as a growth plate cartilage-derived secretory protein and identified as a novel adipokine [2]. CTRP3 mRNA was detected in various tissues, and the protein is found *in vivo* in serum, adipose, muscle, liver, kidney, lung and spleen [2,3,4,5]. Because of its similar structure to adiponectin, its role in the regulation of inflammation, glucose metabolism and lipid metabolism was pursued in most studies. CTRP3 reduces the secretion of pro-inflammatory cytokine IL-6 and TNF-α, without affecting IL-10 synthesis, in response to lipopolysaccharide (LPS) stimulation in both monocytes and adipocytes [6]. In human colonic fibroblasts, CTRP3 significantly inhibited LPS-induced IL-8 release without affecting IL-6 and TNF, lowered TGFβ levels in the supernatants of these cells, and reduced connective tissue growth factor expression [7]. CTRP3 regulated hepatic glucose output and attenuated diet-induced hepatic steatosis by regulating triglyceride metabolism [8,9]. Furthermore, CTRP3 was shown to be an important cytokine which plays crucial roles in regulating the growth of diverse types of cells, for instance, chondrogenic precursor cells [10], endothelial cells [11], osteosarcoma cells [12], vascular smooth cells [13] and cardiomyocytes [14]. CTRP3 also plays a protective role in cardiac infarction through anti-apoptosis and pro-angiogenesis effects in cardiomyocytes [14]. Therefore, by participating in adipokine secretion, fatty acid oxidation, inflammation, cell proliferation, differentiation and apoptosis, CTRP3 has broad functions in regulating various biological processes.

Though CTRP3 is acknowledged as a novel cytokine, its other functions in metabolism and endocrine are still unknown. Based on the findings that CTRP3 stimulates proliferation and anti-apoptosis of several types of cells, in this study, we investigated the potential functions of CTRP3 in regulating cell growth, differentiation and apoptosis of prostate cells.

## Materials and Methods

### Cell culture

The human prostate epithelial cell line RWPE-1 was purchased from the American Type Culture Collection (ATCC Number CRL-11609). RWPE-1 cells were maintained in keratinocyte serum-free medium (KSFM; GIBCO Laboratories, Grand Island, NY) supplemented with 50 mg/L bovine pituitary extract, 5% l-glutamine and 5 μg/L EGF. RWPE-1 cells were maintained in a humidified incubator (5% CO_2_) at 37°C.

Cells were treated with various concentrations of CTRP3 as indicated and analyzed as described below. In the control experiments, 0.1 M phosphate buffer (pH 7.2) containing 0.1% gelatin and 40% glycerol was added to the culture.

To detect the effects of a PKC inhibitor, staurosporine (Santa Cruz, CA, USA), on CTRP3-induced proliferation, anti-apoptosis and change of cell cycle, RWPE-1 cells were pretreated with 0.25μmol/L of staurosporine before and during the stimulation with 10 μg/mL CTRP3.

### Antibodies

Monoclonal anti-FLAG M2 antibody was purchased from Sigma-Aldrich (St. Louis, MO, MA USA). Goat anti-human CTRP3 antibody was purchased from Abcam (ab36870, Cambridge, MA USA). GLRX3, DDAH1 and 14-3-3 sigma antibodies were purchased from Santa Cruz Biotechnology (Santa Cruz, CA, USA). Phospho-(ser) PKC substrate antibody 2261 was purchased from Cell Signaling Technology (CST, MA, USA).

### Generation and purification of CTRP3

A pcDNA3.1 expression construct encoding a C-terminal FLAG-tagged CTRP3 was used to generate CTRP3 protein. HEK 293T cells were cultured in FreeStyle293 expression medium, then transfected using 293fectin (Invitrogen) according to the manufacturer’s instructions. Four days later, an anti-FLAG affinity gel (Sigma-Aldrich) was used to purify the supernatants. Purified protein was dialyzed against 20 mmol/L HEPES buffer (pH 8.0).

### Western blot analysis

Prostate cancer tissue was used as a positive control in western blot analysis of purified CTRP3 protein. The specimen was collected from a prostate cancer patient confirmed by pathology. This study was ratified by the ethics committees of Central Hospital of Longgang District. The piece of tissue was crushed in liquid nitrogen, and then used to extract protein. Total cellular proteins were prepared from treated or untreated cells by lysing cells in lysis buffer (CelLytic, Sigma-Aldrich). Lysates were resolved on 10% or 12.5% polyacrylamide gels. Proteins were transferred to a PVDF membrane (Bio-Rad, Hercules, CA, USA), which was blocked with 5% milk containing TBS-T buffer (0.05% Tween-20) and incubated with primary antibodies at 4°C overnight. After washes with TBST, the membranes were incubated with horseradish peroxidase-conjugated goat anti-rabbit IgG, donkey anti-goat IgG, or goat anti-mouse IgG antibodies (Jackson, USA). Immunoreactive bands were visualized with the Western Breeze chromogenic detection system (Invitrogen).

### Cell proliferation assay

RWPE-1 cells were seeded in a 96-well plate at a density of 1 x 10^3^ cells/well and treated with various concentrations of CTRP3 (3 μg/mL, 10 μg/mL and 30 μg/mL) in triplicate at 37°C, with 5% CO_2_. After incubation, cell proliferation was determined by the MTT assay. MTT (20 μl; 5 mg/mL PBS, pH 7.4; Sigma-Aldrich) was added to the cells for 4 h. Then, MTT-containing medium was removed and 100 μL dimethyl sulfoxide (Sigma-Aldrich) was added to each well for 10 min. The optical density (OD) of the samples was measured at an absorbance of 490 nm (Epoch 2; BioTek Instruments, Inc., Winooski, VT, USA).

### Analysis of apoptosis by flow cytometry and TUNEL staining

The number of apoptotic cells was measured by flow cytometry using the Annexin V-FITC apoptosis detection kit (Roche, Germany). Approximately 1–5 x 10^5^ cells were acquired in each sample. Samples were analyzed by bivariate flow cytometry using a BD FACSCanto cytometer equipped with FlowJo software.

TUNEL staining was performed using the In Situ Cell Death Detection Kit-TMR red (Roche, Germany) following the manufacturer’s instructions. Both TMR (red) and DAPI (blue) fluorescence were visualized and imaged using an IX53 inverted phase contrast microscope (OLYMPUS Inc., Japan).

### Analysis of cell cycle by flow cytometry

RWPE-1 cells were harvested by trypsin-EDTA and fixed in ethanol (70% in PBS). At least 1 to 2 h before flow cytometry, cells were resuspended and then stained with 10 μg/mL RNase A and 5 μg/mL propidium iodide in PBS. Cell distribution in the different phases of the cell cycle was analyzed by flow cytometry (FACSCalibur-S System, BD Bioscience) equipped with FlowJo software.

### Two-dimensional polyacrylamide gel electrophoresis (2D-PAGE)

Cells (2.5 × 10^7^ cells) were lysed in 100 μL cell lysis buffer (C2360, CelLytic, Sigma-Aldrich). The samples were placed at room temperature for 60 min and then centrifuged for 20 min at 4°C, 14,000 rpm. The supernatant was harvested and proteins were collected and stored at -80°C. For first D electrophoresis, the protein samples mixed with 350 μL rehydration buffer were loaded onto each IPG strip in a 2D gel system. Then, strips were transferred with 12% SDS-polyacrylamide gel on the Amersham Dalt II system.

### Mass spectrometry

Protein identification via mass spectrometric peptide sequencing was conducted at an 4700 Proteomics Analyzer using MALDI TOF/TOF (Applied Biosystems, Foster City, CA). The resulting MS and MS/MS peptides were matched to the Swiss-Prot and NCBI protein databases.

### Statistical analysis

Data were expressed as mean ± SE and evaluated statistically using t-test and repeated measures ANOVA with SPSS (version 19.0) software. A value of P < 0.05 was considered statistically significant.

## Results

### Generation and confirmation of CTRP3

**Fig 1:**
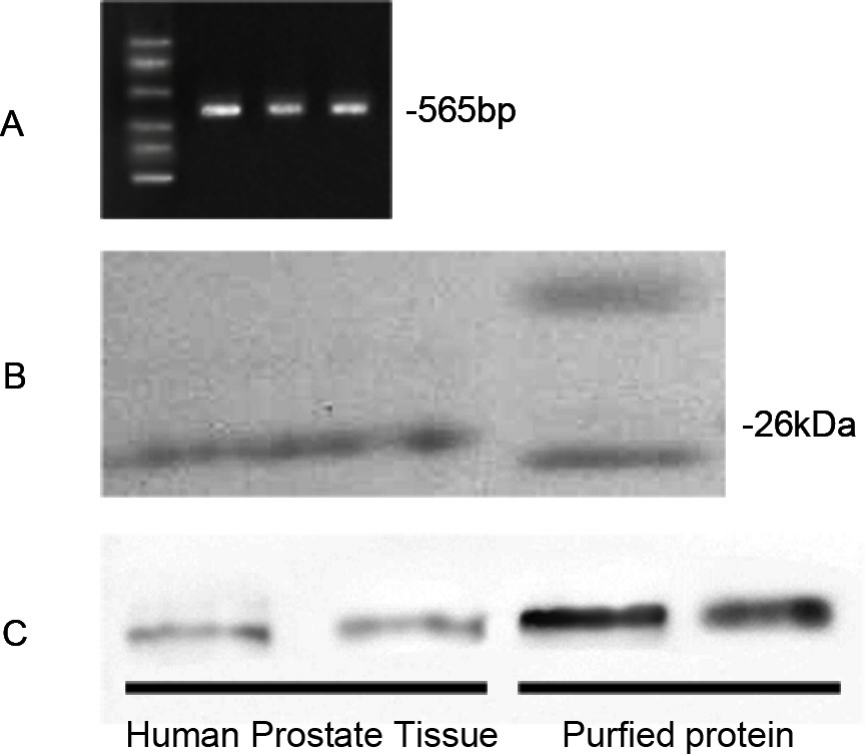
Generation and confirmation of CTRP3. (A) RT-PCR analysis of the pcDNA3.1 expression construct encoding a C-terminal FLAG-tagged CTRP3. (B) Western blot analysis of HEK 293T cells expressing ectopic CTRP3 protein. (C) Western blot analysis of purified CTRP3 protein. CTRP3 protein expressed in prostate cancer tissue was used as a positive control. Lane 1 and Lane 2 were the results from the same sample.

To generate purified CTRP3 protein, we transiently transfected HEK 293T cells with a pcDNA3.1 expression construct encoding a C-terminal FLAG-tagged CTRP3 (Fig 1A and 1B), and then purified the fusion protein using anti-FLAG affinity gel. The purified FLAG-tagged protein was confirmed by western blot analysis (Fig 1C).

### CTRP3 promotes the proliferation of prostate cells

**Fig 2:**
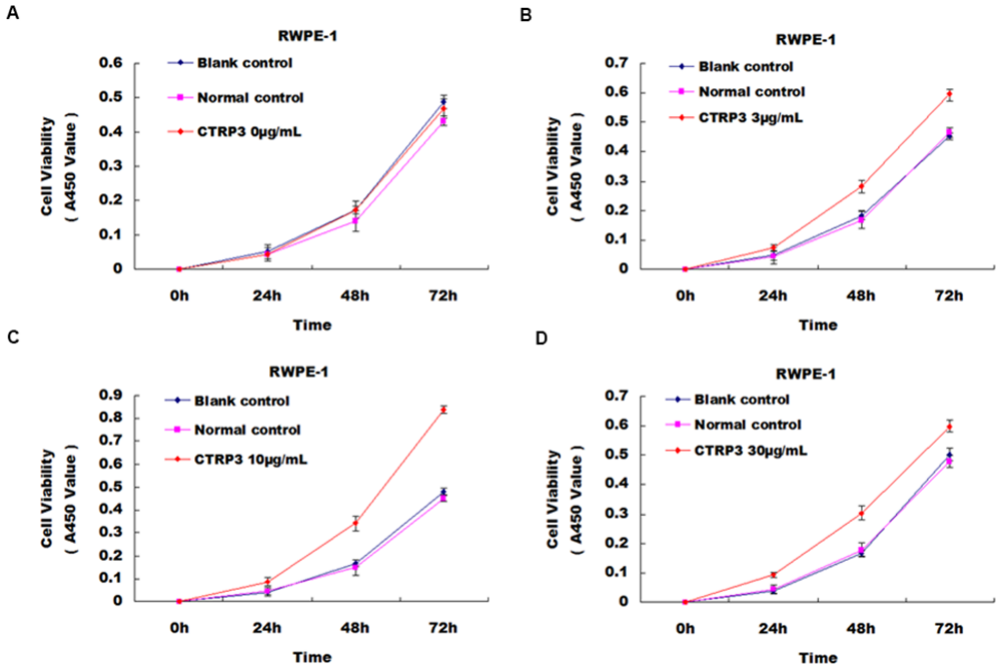
Growth curve demonstrating the effects of CTRP3 on the proliferation of prostate cells. Cells were treated with various concentrations of CTRP3: (A) 0 μg/mL, (B) 3 μg/mL, (C) 10 μg/mL, (D) 30 μg/mL. The optimum concentration of CTRP3 for the growth of RWPE-1 was 10 μg/mL.

Previous studies showed that CTRP3 could promote proliferation of several types of cells. Thus, we first determined whether CTRP3 also could promote the proliferation of RWPE-1 prostate cells. We evaluated the number of cells treated with different concentrations of CTRP3 (0, 3, 10, 30 μg/mL) for different incubation times (0, 24, 48, 72 h). Compared with the control group, the numbers of RWPE-1 cells treated with 3 μg/mL, 10 μg/mL and 30 μg/mL CTRP3 were increased by 62.75%, 89.07%, 106.98% at 24 h, respectively; 68.32%, 104.05%, 80.24% at 48 h; and 28.64%, 80.25%, 28.64% at 72 h (all *P* < 0.01). We observed the largest effect of CTRP3 in promoting cell proliferation upon treatment with 10 μg/mL CTRP3 for 72 h. The differences between 10 μg/mL and 3 μg/mL, and 10 μg/mL and 30 μg/mL treatment groups at both 48 h and 72 h were also significant (all *P* < 0.05). Together these results indicate that 10 μg/mL was the optimal concentration for CTRP3 in promoting proliferation of RWPE-1 prostate cells (Fig 2).

### Effect of CTRP3 on apoptosis of prostate cells

**Fig 3:**
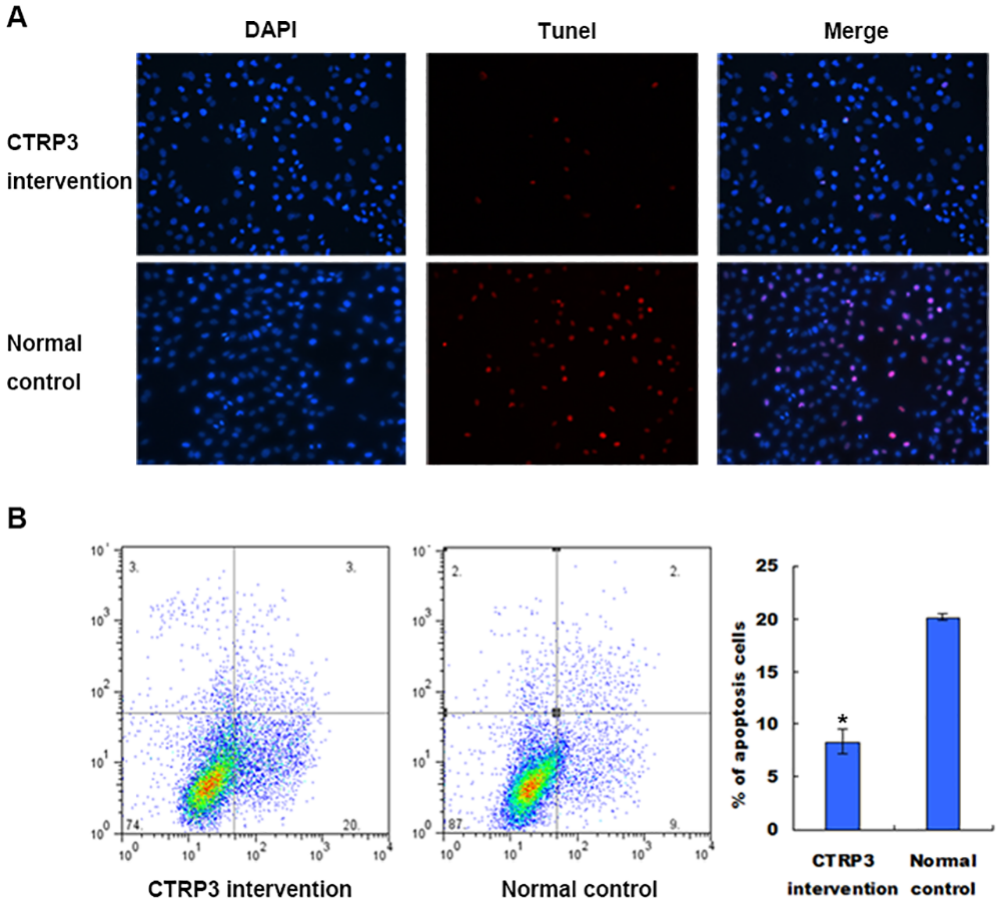
Effect of CTRP3 on the apoptosis of prostate cells. (A) TUNEL-positive cells among DAPI-positive cells were decreased in cells treated with CTRP3. TUNEL-positive apoptotic nuclei and DAPI-stained nuclei were visualized at ×200 magnification. (B) Flow cytometry analysis demonstrated a significantly decreased ratio of apoptotic cells in cells treated with CTRP3. Data are representative of three independently performed experiments. Mean±SD. *P < 0.01, as compared with the control group.

The above results showed that the maximal effect of stimulating cell proliferation occurred with 10 μg/mL CTRP3 in RWPE-1 prostate cells for 72 h. We next investigated whether CTRP3 protected RWPE-1 cells from apoptosis to promote cell cycle progression under these conditions by TUNEL staining and flow cytometry analyses. We observed a decreased number of TUNEL-positive cells among the DAPI-positive cells in cells treated with CTRP3 (Fig 3A). Flow cytometry analysis showed that CTRP3 effectively blocked the apoptotic process, with a significant decreased ratio of apoptotic cells (treated cells, 8.34±1.175 vs. control, 20.163±0.35; *P* < 0.01) (Fig 3B).

### Differentially expressed proteins in prostate cells treated by CTRP3

**Fig 4:**
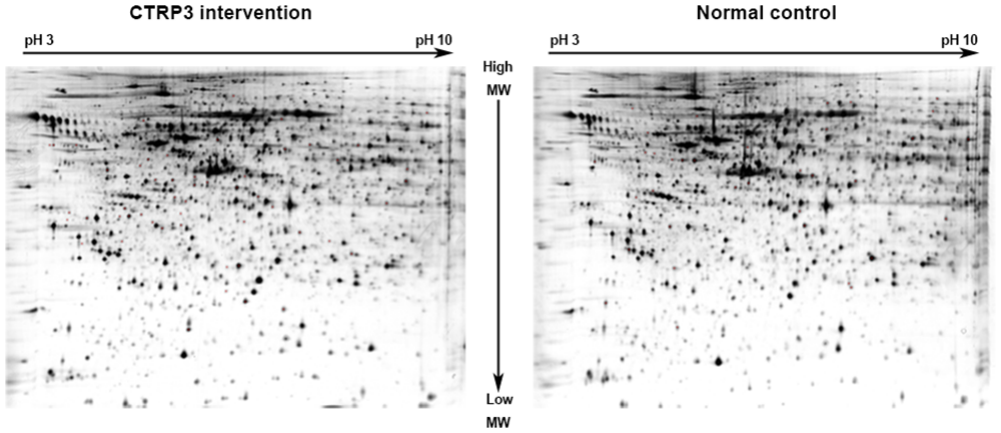
2-dimensional polyacrylamide gel electrophoresis maps of prostate cells in CTRP3 treated cells compared with control cells. Data are representative of three independent performed experiments.

**Fig 5:**
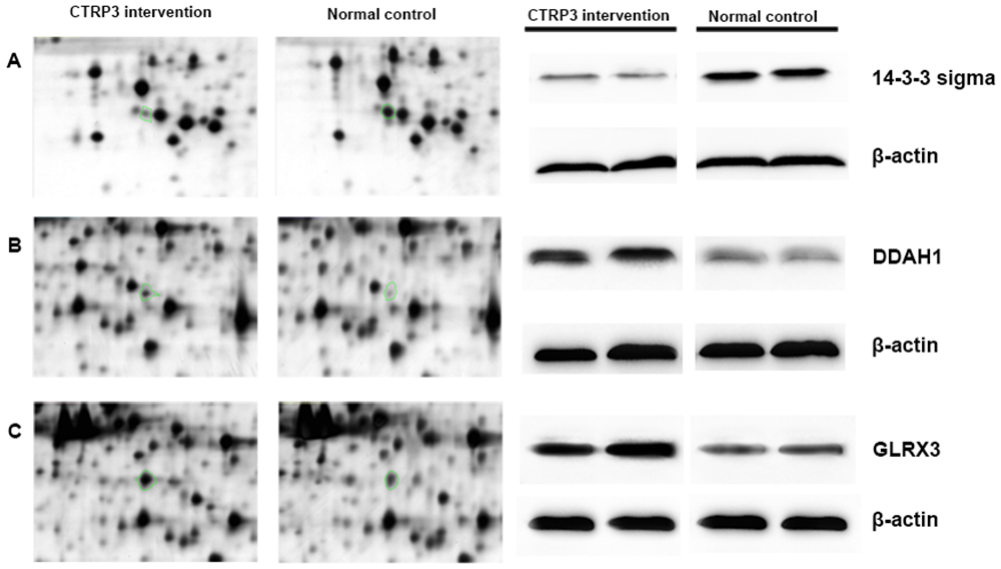
Cropped 2D gel images and western blot analyses of selected proteins. (A) 14-3-3 sigma, (B) DDAH1, (C) GLRX3.

We next sought to identify differentially expressed proteins in CTRP3-treated prostate cells. We performed 2D-PAGE analyses with lysates from RWPE-1 cells treated with 10 μg/mL CTRP3 for 72 h and control cells, using three pairs of samples. Overall, the protein spot patterns of individual samples were mostly consistent, indicating no heterogeneity of each sample (Fig 4). Nevertheless, the gels also showed significantly different protein expression for each pair of samples. We focused on the protein spots that showed a distinctly different expression and identified the spots using mass spectrometry technique. Five prominent spots were identified as cytokeratin-19, GLRX3, DDAH1, cytokeratin-17 and 14-3-3 sigma (Table 1). Cytokeratin-19, GLRX3 and DDAH1 were upregulated proteins in CTRP3-treated cells, and cytokeratin-17 and 14-3-3 sigma were downregulated proteins. Among these significantly differentially expressed proteins, we confirmed GLRX3, DDAH1 and 14-3-3 sigma expression in CTRP3-treated cells using western blot analysis (Fig 5).

**Table 1:** Identification of protein spots based on MS analysis.

	Peptide sequence	Protein
Down-regulated	TKFETEQALR LSVEADINGL RRVLDELTLA	keratin 17
	AEQAERYEDM AAFMKGAVEK GEE	14-3-3 Sigma
Up-regulated	QSS ATSSFGGLGG GSVR	keratin 19
	HA SSGSFLSSAN EHLKEDLNLR	GLRX3
	ATHAVVRALPESLGQHALRSAKGEEVDVAR	DDAH1

### Effect of CTRP3 on proliferation, apoptosis and cell cycle is mediated through PKC signaling pathway

**Fig 6:**
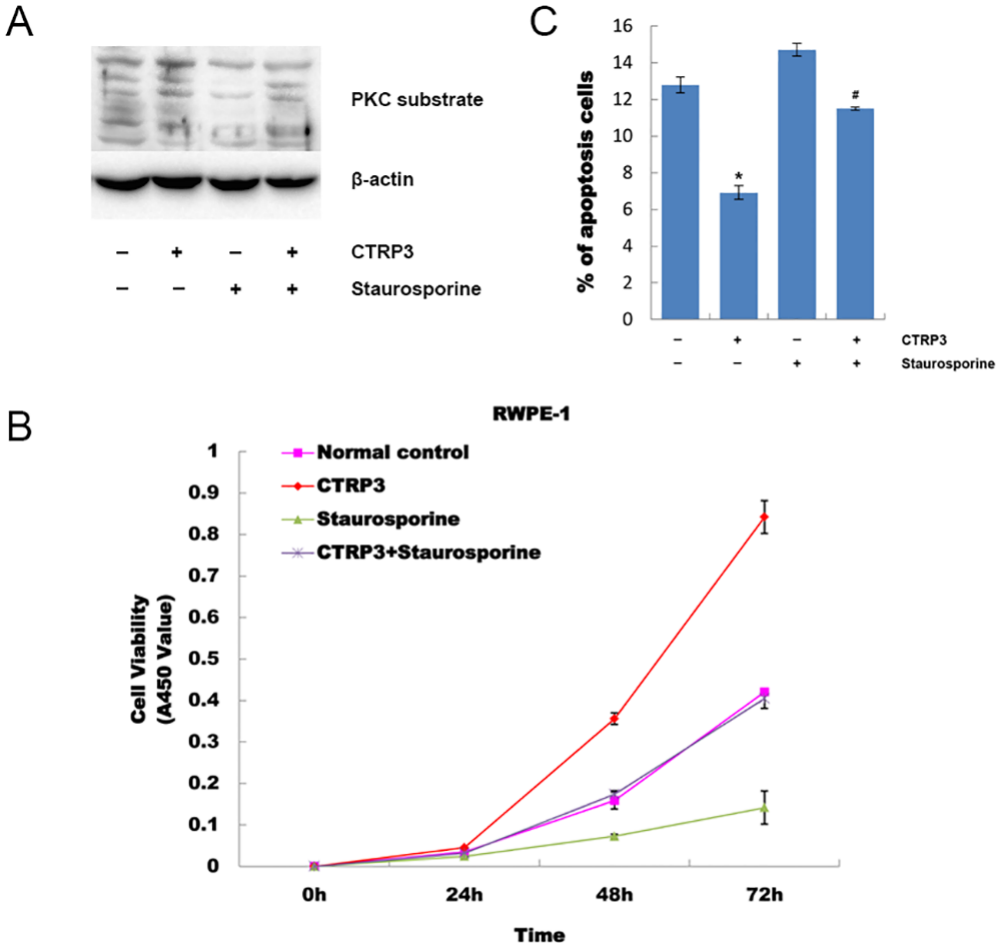
Effect of CTRP3 on proliferation and apoptosis was mediated through the PKC signaling pathway. (A) Phosphorylation of intracellular PKC substrates increased in CTRP3 treated RWPE-1 cells and went back to normal when pretreated with PKC inhibitor. (B) RWPE-1 cells were treated with 10 μg/mL of CTRP3 or staurosporine and then analyzed by a MTT assay. PKC inhibitor staurosporine completely abolished the CTRP3-stimulated proliferation in RWPE-1 cells. **(C)** RWPE-1 cells were treated with 10 μg/mL of CTRP3 or staurosporine and then analyzed by flow cytometry. PKC inhibitor staurosporine completely abolished the CTRP3-stimulated anti-apoptosis effect. Mean±SD. *P < 0.05, as compared with the control group; #*P* < 0.05, as compared with the CTRP3-treated group.

**Fig 7:**
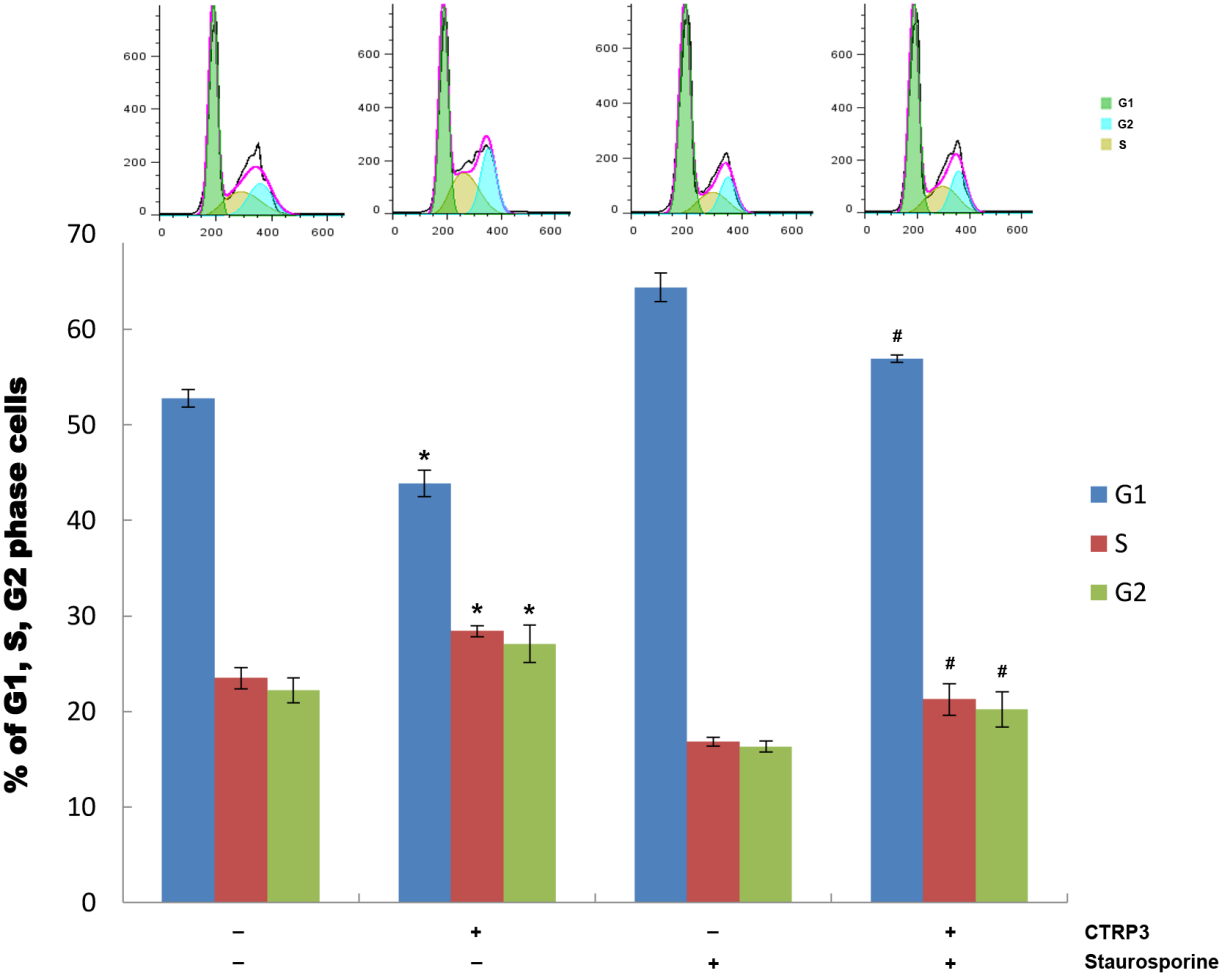
Effect of CTRP3 on cell cycle was mediated through the PKC signaling pathway. RWPE-1 cells were treated with 10 μg/mL of CTRP3 or staurosporine and then analyzed by flow cytometry. The percent of RWPE-1 cells in the G1 phase decreased significantly upon CTRP3 treatment whereas the percentage of cells in the S and G2 phase increased. PKC inhibitor staurosporine completely abolished the CTRP3-stimulated effect. Mean±SD. *P < 0.05, as compared with the control group; #*P* < 0.05, as compared with the CTRP3-treated group.

The above results strongly indicated that CTRP3 stimulated proliferation and anti-apoptosis of prostate cells through PKC signaling pathways. To further confirm that, PKC activity in CTRP3 treated RWPE-1 cells was inhibited using a PKC inhibitor, staurosporine. Western blot analysis showed that the phosphorylation of intracellular PKC substrates increased in CTRP3 treated RWPE-1 cells and went back to normal when pretreated with PKC inhibitor (Fig 6A). MTT and flow cytometry analysis showed that the stimulatory effects of CTRP3 on proliferation and anti-apoptosis were completely blocked by staurosporine (Fig 6B, Fig 6C). Flow cytometry analysis also showed a significant decrease of cells in the G1 phase and an increase of cells in the S and G2 phase upon CTRP3 treatment (treated cells, 42.85±1.40 vs. control, 52.77±0.90; 28.41±0.57 vs. 23.49±1.13; 27.08±1.97 vs. 22.20±1.32, respectively) (all P < 0.05) (Fig 7). The changes in the cell cycle induced by CTRP3 were also reversed by staurosporine. These results suggest the involvement of the PKC pathway in the ability of CTRP3 to stimulate proliferation and anti-apoptosis in prostate RWPE-1 cells.

## Discussion

CTRP3 and adiponectin have highly homologous structures, which is a characteristic of the CTRP family. While structurally related to the other 15 members of the CTRP family, CTRP3 emerges as a novel adipokine with potential functions in the regulation of glucose metabolism and lipid metabolism. However, some studies also showed that CTRP3 is related with tumor development [12]. CTRP3 also plays an important role in the proliferation and anti-apoptosis in several cells. However, little is known about its other biological activities.

In the present study, we provide the first data on the biological effects of CTRP3 in prostate cells. We showed that CTRP3 promoted RWPE-1 prostate cell proliferation in a concentration-dependent and time-dependent manner. We found that the most effective concentration in inducing proliferation of RWPE-1 cells was 10 μg/mL. In addition, upon treatment of RWPE-1 cells with 10 μg/mL CTRP3, we found that the levels of apoptotic cells significantly decreased. Therefore, our results show that CTRP3 could stimulate proliferation and anti-apoptosis activity in prostate cells.

So far, little is known about CTRP3-specific receptor or signaling pathways. Hou *et al*. showed that CTRP3 protected bone marrow derived mesenchymal stem cells from hypoxia/SD-induced apoptosis through the PI3K/Akt signaling pathway [15]. Yi *et al*. showed that CTRP3 significantly increased Akt phosphorylation in mouse cardiac myocytes [14]. However, CTRP3 had no significant effect on cardiac AMP-activated protein kinase phosphorylation [14]. A Japanese group discovered that CTRP3 could also activate ERK1/2 (extracellular signal-regulated kinase 1/2) and p38 MAPK (mitogen-activated protein kinase) signaling pathways to promote the proliferation of vascular smooth muscle cells [13] and mouse endothelial MSS31 cells [11]. CTRP3 also activated only ERK1/2 to promote the proliferation of osteosarcoma cells [12] and migration of mouse endothelial MSS31 cells [11]. The same group also discovered that CTRP3 activated ERK1/2 and PI3K/Akt, but neither c-jun N-terminal kinase (JNK) nor p38 MAPK signaling pathways, to stimulate proliferation of mesenchymal chondroprogenitor cells [16]. Otani *et al*. also reported that the stimulatory effect of CTRP3 on testosterone production was associated with activation of the cAMP/PKA signaling pathway [17]. These recent studies highlighted the complexity of the effects and mechanisms of signaling pathways activated by CTRP3 in diverse cells.

2D gel electrophoresis has been developed as a standardized and widely-used technique, thus offering new opportunities for pathways detection. Its advantages include high resolving power and the ability to display several thousand proteins at once. Because of these advantages, we used 2D gel electrophoresis to explore the possible mechanisms of CTRP3 impact on the prostate cells. We identified three proteins (cytokeratin-19, GLRX3, DDAH1) that showed upregulated expression in response to CTRP3 and two proteins (cytokeratin-17 and 14-3-3 sigma) that were downregulated. We focused on GLRX3, DDAH1 and 14-3-3 sigma, as these proteins exhibit specific functions in signaling pathways, and confirmed the 2D results using western blot analysis.

14-3-3 sigma is an important regulator involved in signaling transduction, stress response, apoptosis, transcriptional regulation and coordination of cell adhesion and motility [18]. The 14-3-3 proteins bind to PKC epsilon to lock it in an open active conformation and downregulate PKC signaling pathways [19]. In addition, 14-3-3 sigma is a p53 target gene and the 14-3-3 sigma protein might act in a positive feedback loop on p53 [20]. Previous studies have identified considerable cross-talk between p53/14-3-3 sigma and PKC signaling pathways. When the p53/14-3-3 sigma signaling pathway is inhibited, PKC signaling pathway is enhanced. 14-3-3 sigma is also involved in various types of cancers, such as lung cancer and prostate cancer [21,22]. By mediating the tumor-suppressive effects of the p53/14-3-3 sigma signaling pathway, 14-3-3 sigma was generally considered as a tumor suppressor protein. In addition, 14-3-3 proteins are integral components of several checkpoints, with a key role in cell cycle regulation [23].

GLRX3, also known as PICOT (PKC-interacting cousin of thioredoxin), is a member of the glutaredoxin family, which includes oxidoreductase enzymes that reduce a variety of substrates using glutathione as a cofactor. GLRX3 contains an N-terminal thioredoxin homology domain that binds and modulates the function of PKC theta [24]. In addition, overexpression of the GLRX3 gene was detected in breast cancer, colon and lung carcinoma, and thus it may serve as a marker for cancer [25,26]. GLRX3 also stimulates breast cancer cell growth and metastasis through redox homeostasis and NF-κB signaling [25]. DDAH1, a member of the dimethylarginine dimethylaminohydrolase (DDAH) family, plays a role in nitric oxide (NO) generation by eliminating cellular concentrations of asymmetric dimethylarginine (ADMA), which in turn inhibits nitric oxide synthase (NOS) activity [27]. DDAH1 regulates endothelial cell proliferation by degrading ADMA, thereby activating the NO/cGMP/PKG pathway [28]. In addition, proteomic analysis of prostate biopsies to distinguish hyperplasia and cancer also found upregulation of DDAH1 in prostate cancer [29].

Members of the PKC family of intracellular serine/threonine kinases play key roles in the regulation of cellular differentiation and proliferation in diverse cell types and in response to varied cytokines and hormones. Both 14-3-3 sigma and GLRX3 identified in this study play a part in PKC signaling pathways. Furthermore, 14-3-3 sigma, which exhibits inhibitory effects in PKC signaling pathways, was downregulated in response to CTRP3, and GLRX3, which is a direct regulator in PKC signaling pathways, was upregulated. The pattern of differential protein expression strongly indicates that CTRP3 stimulates proliferation and anti-apoptosis of prostate cells through PKC signaling pathways. In the present study, CTRP3 could increase phosphorylation of intracellular PKC substrates in RWPE-1 cells. Moreover, inhibition of PKC activity by a PKC inhibitor staurosporine completely abolished the increased phosphorylation of intracellular PKC substrates and CTRP3-stimulated effect in RWPE-1 cells. Thus, our findings that the stimulatory effect of CTRP3 on proliferation and anti-apoptosis is mediated through PKC signaling pathway is undoubted. In addition, 14-3-3 sigma, GLRX3 and DDAH1 have been shown to function in various kinds of tumors as well as prostate cancer. Together this suggests that CTRP3 may promote the transformation from prostate cells to cancer cells.

In summary, our data support CTRP3 as a novel cytokine for stimulating proliferation and anti-apoptosis of prostate cells through PKC signaling pathways. Furthermore, the potential involvement between CTRP3 and prostate cancer may provide new insights into the molecular mechanism underlying prostate cancer.

## Supporting Information

S1 Fig Raw data of flow cytometry about the anti-apoptosis effects of CTRP3. (PDF)

S1 Table OD values of RWPE-1 cells with different treatment. (XLSX)

## References

[1] Schaffler A, Buechler C. CTRP family: linking immunity to metabolism. Trends Endocrinol Metab.2012; 23: 194–204. 10.1016/j.tem.2011.12.003 22261190

[2] Maeda T, Abe M, Kurisu K, Jikko A, Furukawa S. Molecular cloning and characterization of a novel gene, CORS26, encoding a putative secretory protein and its possible involvement in skeletal development. J Biol Chem.2001; 276: 3628–3634. 11071891 10.1074/jbc.M007898200

[3] Schaffler A, Ehling A, Neumann E, Herfarth H, Paul G, Tarner I, et al Genomic organization, promoter, amino acid sequence, chromosomal localization, and expression of the human gene for CORS-26 (collagenous repeat-containing sequence of 26-kDa protein). Biochim Biophys Acta.2003; 1630: 123–129. 14654242 10.1016/j.bbaexp.2003.08.013

[4] Yoo HJ, Hwang SY, Hong HC, Choi HY, Yang SJ, Choi DS, et al Implication of progranulin and C1q/TNF-related protein-3 (CTRP3) on inflammation and atherosclerosis in subjects with or without metabolic syndrome. PLoS One.2013; 8: e55744 10.1371/journal.pone.0055744 23409033 PMC3567086

[5] Weigert J, Neumeier M, Schaffler A, Fleck M, Scholmerich J, Schütz C, et al The adiponectin paralog CORS-26 has anti-inflammatory properties and is produced by human monocytic cells. FEBS Lett.2005; 579: 5565–5570. 16213490 10.1016/j.febslet.2005.09.022

[6] Kopp A, Bala M, Buechler C, Falk W, Gross P, Neumeier M, et al C1q/TNF-related protein-3 represents a novel and endogenous lipopolysaccharide antagonist of the adipose tissue. Endocrinology.2010; 151: 5267–5278. 10.1210/en.2010-0571 20739398

[7] Hofmann C, Chen N, Obermeier F, Paul G, Buchler C, Kopp A, et al C1q/TNF-related protein-3 (CTRP-3) is secreted by visceral adipose tissue and exerts antiinflammatory and antifibrotic effects in primary human colonic fibroblasts. Inflamm Bowel Dis.2011; 17: 2462–2471. 10.1002/ibd.21647 21351204

[8] Peterson JM, Wei Z, Wong GW. C1q/TNF-related protein-3 (CTRP3), a novel adipokine that regulates hepatic glucose output. J Biol Chem.2010; 285: 39691–39701. 10.1074/jbc.M110.180695 20952387 PMC3000950

[9] Peterson JM, Seldin MM, Wei Z, Aja S, Wong GW. CTRP3 attenuates diet-induced hepatic steatosis by regulating triglyceride metabolism. Am J Physiol Gastrointest Liver Physiol.2013; 305: G214–G224. 10.1152/ajpgi.00102.2013 23744740 PMC3742855

[10] Maeda T, Jikko A, Abe M, Yokohama-Tamaki T, Akiyama H, Furukawa S, et al Cartducin, a paralog of Acrp30/adiponectin, is induced during chondrogenic differentiation and promotes proliferation of chondrogenic precursors and chondrocytes. J Cell Physiol.2006; 206: 537–544. 16155912 10.1002/jcp.20493

[11] Akiyama H, Furukawa S, Wakisaka S, Maeda T. CTRP3/cartducin promotes proliferation and migration of endothelial cells. Mol Cell Biochem.2007; 304: 243–248. 17534697 10.1007/s11010-007-9506-6

[12] Akiyama H, Furukawa S, Wakisaka S, Maeda T. Elevated expression of CTRP3/cartducin contributes to promotion of osteosarcoma cell proliferation. Oncol Rep.2009; 21: 1477–1481. 19424626 10.3892/or_00000377

[13] Maeda T, Wakisaka S. CTRP3/cartducin is induced by transforming growth factor-beta1 and promotes vascular smooth muscle cell proliferation. Cell Biol Int.2010; 34: 261–266. 10.1042/CBI20090043 19947921

[14] Yi W, Sun Y, Yuan Y, Lau WB, Zheng Q, Wang X, et al C1q/tumor necrosis factor-related protein-3, a newly identified adipokine, is a novel antiapoptotic, proangiogenic, and cardioprotective molecule in the ischemic mouse heart. Circulation.2012; 125: 3159–3169. 10.1161/CIRCULATIONAHA.112.099937 22653084 PMC3391311

[15] Hou M, Liu J, Liu F, Liu K, Yu B. C1q tumor necrosis factor-related protein-3 protects mesenchymal stem cells against hypoxia- and serum deprivation-induced apoptosis through the phosphoinositide 3-kinase/Akt pathway. Int J Mol Med.2014; 33: 97–104. 10.3892/ijmm.2013.1550 24212403

[16] Akiyama H, Furukawa S, Wakisaka S, Maeda T. Cartducin stimulates mesenchymal chondroprogenitor cell proliferation through both extracellular signal-regulated kinase and phosphatidylinositol 3-kinase/Akt pathways. FEBS J.2006; 273: 2257–2263. 16650001 10.1111/j.1742-4658.2006.05240.x

[17] Otani M, Kogo M, Furukawa S, Wakisaka S, Maeda T. The adiponectin paralog C1q/TNF-related protein 3 (CTRP3) stimulates testosterone production through the cAMP/PKA signaling pathway. Cytokine.2012; 58: 238–244. 10.1016/j.cyto.2012.01.018 22342437

[18] Hermeking H, Benzinger A. 14-3-3 proteins in cell cycle regulation. Semin Cancer Biol.2006; 16: 183–192. 16697662 10.1016/j.semcancer.2006.03.002

[19] Saurin AT, Durgan J, Cameron AJ, Faisal A, Marber MS, Parker PJ. The regulated assembly of a PKCepsilon complex controls the completion of cytokinesis. Nat Cell Biol.2008; 10: 891–901. 10.1038/ncb1749 18604201

[20] Rohaly G, Chemnitz J, Dehde S, Nunez AM, Heukeshoven J, Deppert W, et al A novel human p53 isoform is an essential element of the ATR-intra-S phase checkpoint. Cell.2005; 122: 21–32. 16009130 10.1016/j.cell.2005.04.032

[21] Osada H, Tatematsu Y, Yatabe Y, Nakagawa T, Konishi H, Harano T, et al Frequent and histological type-specific inactivation of 14-3-3sigma in human lung cancers. Oncogene.2002; 21: 2418–2424. 11948426 10.1038/sj.onc.1205303

[22] Lodygin D, Diebold J, Hermeking H. Prostate cancer is characterized by epigenetic silencing of 14-3-3sigma expression. Oncogene.2004; 23: 9034–9041. 15489902 10.1038/sj.onc.1208004

[23] Gardino AK, Yaffe MB. 14-3-3 proteins as signaling integration points for cell cycle control and apoptosis. Semin Cell Dev Biol.2011; 22: 688–695. 10.1016/j.semcdb.2011.09.008 21945648 PMC3507455

[24] Witte S, Villalba M, Bi K, Liu Y, Isakov N, Altman A. Inhibition of the c-Jun N-terminal kinase/AP-1 and NF-kappaB pathways by PICOT, a novel protein kinase C-interacting protein with a thioredoxin homology domain. J Biol Chem.2000; 275: 1902–1909. 10636891 10.1074/jbc.275.3.1902

[25] Qu Y, Wang J, Ray PS, Guo H, Huang J, Shin-Sim M, et al Thioredoxin-like 2 regulates human cancer cell growth and metastasis via redox homeostasis and NF-kappaB signaling. J Clin Invest.2011; 121: 212–225. 10.1172/JCI43144 21123948 PMC3007146

[26] Cha MK, Kim IH. Preferential overexpression of glutaredoxin3 in human colon and lung carcinoma. Cancer Epidemiol.2009; 33: 281–287. 10.1016/j.canep.2009.08.006 19797004

[27] Maas R, Boger R, Luneburg N. ADMA and the role of the genes: lessons from genetically modified animals and human gene polymorphisms. Pharmacol Res.2009; 60: 475–480. 10.1016/j.phrs.2009.07.012 19666122

[28] Zhang P, Hu X, Xu X, Chen Y, Bache RJ. Dimethylarginine dimethylaminohydrolase 1 modulates endothelial cell growth through nitric oxide and Akt. Arterioscler Thromb Vasc Biol.2011; 31: 890–897. 10.1161/ATVBAHA.110.215640 21212404 PMC3064458

[29] Ummanni R, Junker H, Zimmermann U, Venz S, Teller S, Giebel J, et al Prohibitin identified by proteomic analysis of prostate biopsies distinguishes hyperplasia and cancer. Cancer Lett. 2008; 266: 171–185. 10.1016/j.canlet.2008.02.047 18384941

