# Supplementary figures and images for: CTRP3 Stimulates Proliferation and Anti-Apoptosis of Prostate Cells through PKC Signaling Pathways
Source: PLoS One. 2015 Jul 28;10(7):e0134006. doi: 10.1371/journal.pone.0134006 (PMC4517796; doi:10.1371/journal.pone.0134006)

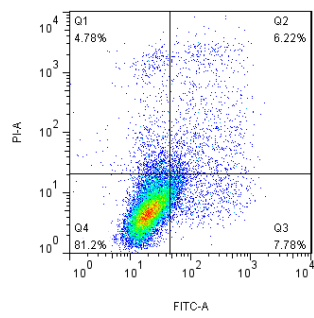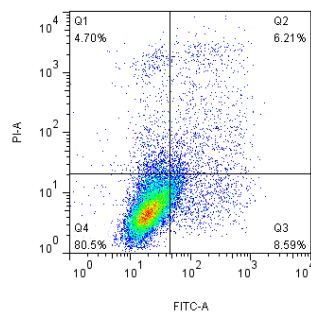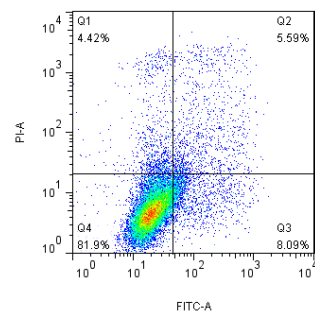

NC

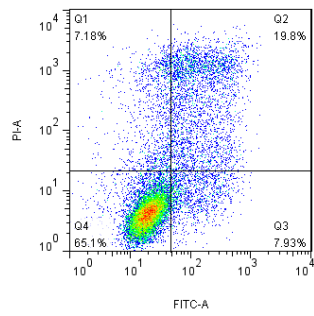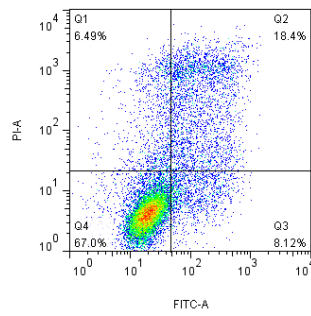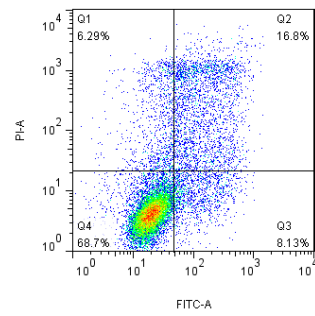

Staurosporine

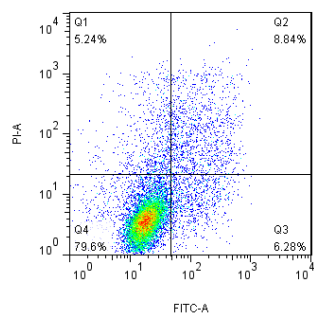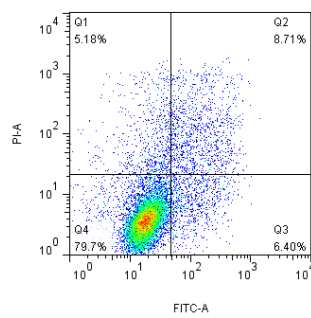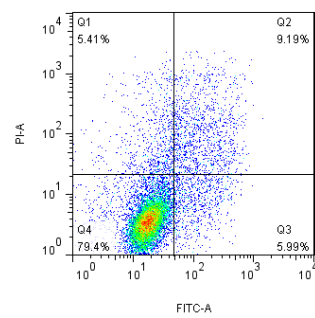

CTRP3+Staurosporine

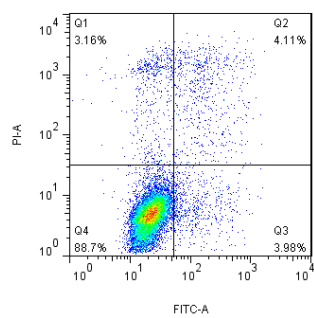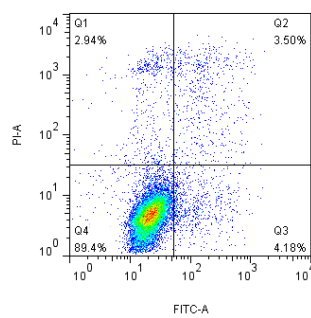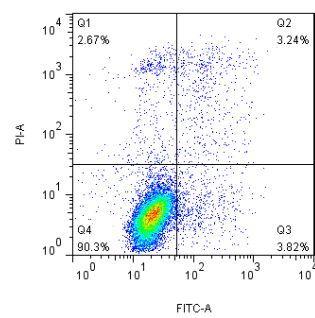

CTRP3

Supplement: S1 Fig — (PDF) [file pone.0134006.s001.pdf]
